# A small-molecular inhibitor against *Proteus mirabilis* urease to treat catheter-associated urinary tract infections

**DOI:** 10.1038/s41598-021-83257-2

**Published:** 2021-02-12

**Authors:** Scarlet Milo, Rachel A. Heylen, John Glancy, George T. Williams, Bethany L. Patenall, Hollie J. Hathaway, Naing T. Thet, Sarah L. Allinson, Maisem Laabei, A. Toby A. Jenkins

**Affiliations:** 1 Department of Chemistry, University of Bath, Bath, BA2 7AY UK; 2 School of Physical Sciences, University of Kent, Canterbury, CT2 7NH UK; 3 Department of Chemistry, Lancaster University, Bailrigg, Lancaster, LA1 4YB UK; 4 Biomedical and Life Sciences Division, Lancaster University, Bailrigg, Lancaster, LA1 4YB UK; 5 Department of Biology and Biochemistry, University of Bath, Bath, BA2 7AY UK

**Keywords:** Clinical microbiology, Biochemistry, Chemical biology, Microbiology, Urology

## Abstract

Infection and blockage of indwelling urinary catheters is significant owing to its high incidence rate and severe medical consequences. Bacterial enzymes are employed as targets for small molecular intervention in human bacterial infections. Urease is a metalloenzyme known to play a crucial role in the pathogenesis and virulence of catheter-associated *Proteus mirabilis* infection. Targeting urease as a therapeutic candidate facilitates the disarming of bacterial virulence without affecting bacterial fitness, thereby limiting the selective pressure placed on the invading population and lowering the rate at which it will acquire resistance. We describe the design, synthesis, and in vitro evaluation of the small molecular enzyme inhibitor 2-mercaptoacetamide (2-MA), which can prevent encrustation and blockage of urinary catheters in a physiologically representative in vitro model of the catheterized urinary tract. 2-MA is a structural analogue of urea, showing promising competitive activity against urease. In silico docking experiments demonstrated 2-MA’s competitive inhibition, whilst further quantum level modelling suggests two possible binding mechanisms.

## Introduction

Catheter-associated urinary tract infections (CAUTIs) are the most common hospital acquired infection, affecting approximately 150–250 million patients globally per year^1^. CAUTIs accounted for 45,717 excess bed days in the UK National Health Service (NHS) hospitals during 2016–2017 period, and cost the NHS between £1.5 and 2.25 billion per year^2,3^. *Proteus mirabilis* (*P. mirabilis*) is the most common cause of bacteremia in nursing homes, bearing a high mortality rate owing to the possibility of progression to sepsis^4,5^. *P. mirabilis* is a Gram-negative, rod-shaped, urease-positive bacteria which forms biofilms on and around the luminal surfaces of the catheter^4^. Urease (a nickel-dependent metalloenzyme) metabolizes urea to a molecule of carbonic acid and two molecules of ammonia (Supplementary Fig. 1)^6^. The primary function of urease expressed by *P. mirabilis* is to provide a nitrogen source for the bacteria^4^. As ammonia is produced, the pH of the urine increases, promoting the formation of struvite (MgNH_4_PO_4_·6H_2_O) and apatite (Ca_10_(PO_4_CO_3_OH)_6_(OH)_2_) crystals^7^. These crystals integrate into the *P. mirabilis* biofilm and attach to the lumen and balloon of the catheter; blocking the flow of the urine down to the drainage bag^8,9^. The resultant extensive crystalline biofilm networks are notoriously difficult to treat, are often antibiotic resistant and may remain within the bladder between catheter changes^10–12^. Catheter blockage causes incontinence and painful distention of the bladder, and can result in urine reflux to the kidneys, causing pyelonephritis and increasing the risk of sepsis^5,13^. Current treatment of blocked catheters generally consists of antibiotic treatment and a catheter change. However, due to struvite and apatite encrustation, removal of blocked catheters is often uncomfortable and painful for the patients. Indeed, the replacement catheter can become rapidly recolonized by bacteria, as it is often placed into the bladder containing a high bacteria titre^5^. Currently, acetohydroxamic acid (AHA) is the only licensed urease inhibitor available for antiureolytic therapy (Supplementary Fig. 2). Having gained FDA approval in 1983 (marketed under the name Lithostat in the USA, and Uronefrex in Kuwait and Spain), it has been widely used to treat hyperammonemia in cirrhosis of patients infected with the urease-positive bacteria *Helicobacter pylori*, as well as chronic urea splitting and recurrent urinary tract infection by *P. mirabilis*^14–17^. However, AHA is known to induce severe side effects including teratogenesis and hemolytic anaemia^15,18–20^. Awareness of such undesirable side effects has resulted in a fall in AHA popularity in recent years, inducing a surge of research into alternative urease inhibitors with less severe toxicological profiles. Targeting urease as a therapeutic candidate facilitates the disarming of bacterial virulence without impacting bacterial viability. Thereby limiting the evolutionary selection pressure placed on the invading bacterial population (since bacteria are not inhibited or destroyed by the therapeutic approach).

**Figure 1:**
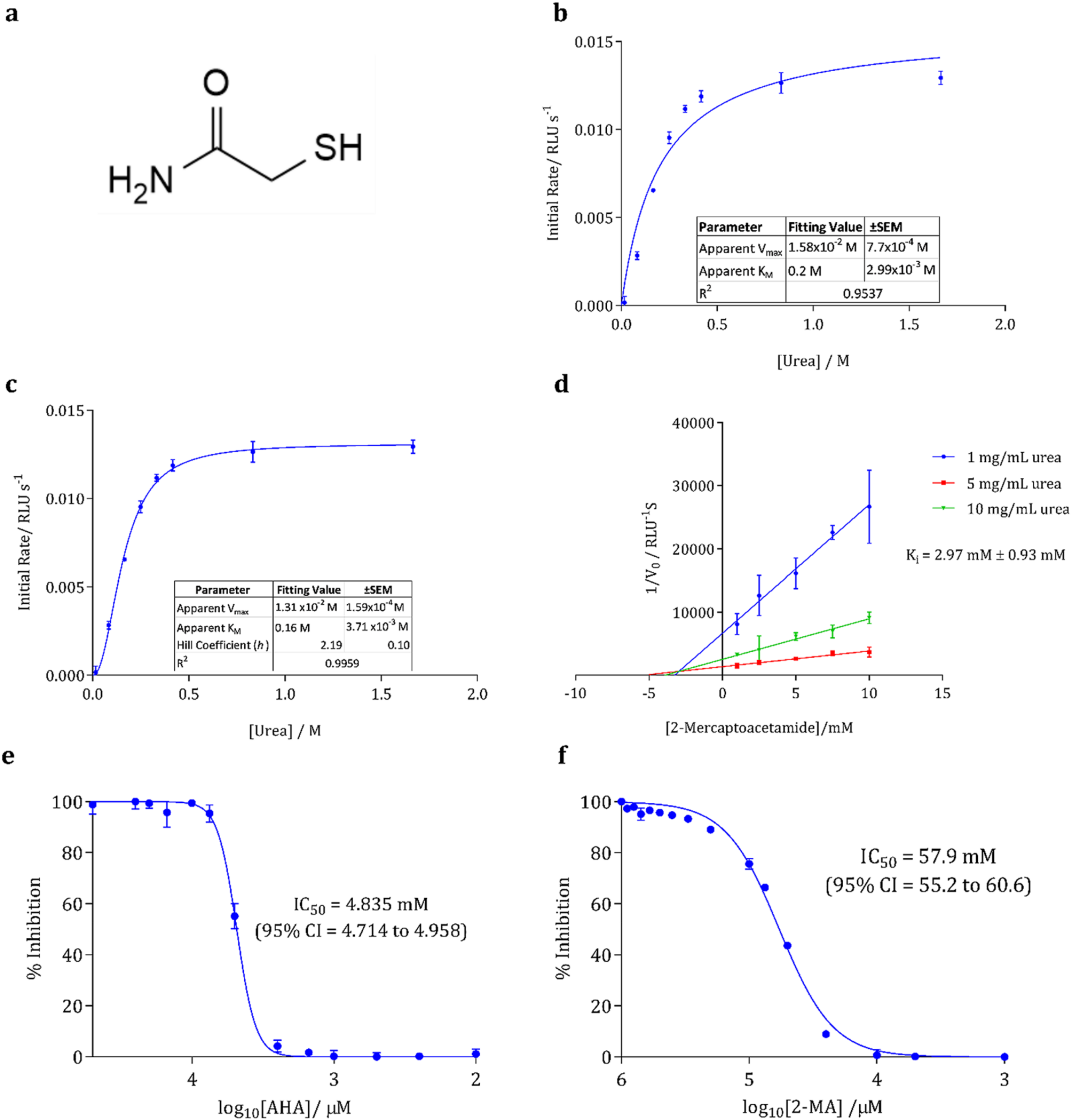
(**a**) Molecular structure of 2-mercatoacetamide, the proposed inhibitor of *P. mirabilis* urease activity. Michaelis–Menten kinetics of *C. ensiformis* urease, with the substrate urea. (**b**) Hyperbolic curve fitting (least squares fit) was used to determine Michaelis–Menten parameters. (**c**) Sigmoidal curve fitting (least squares fit, Hill slope) was used to determine Michaelis–Menten parameters and determination of the Hill Coefficient. (**d**) Dixon plot for the competitive inhibition of *C. ensiformis* urease by 2-MA. The abscissa of line intersection yields the inhibition constant (− K_i_). The error of K_i_ was calculated as relative uncertainty combining the standard error of mean (SEM) error from the equations of each line. Determining IC_50_ of AHA (**e**) and 2-MA (**f**) inhibiting urease. Data was fitted with sigmoidal dose–response curves and IC_50_ determined by non-linear regression. Results are from three biological replicates; error bars represent SEM (standard error of mean). Data fitted using GraphPad Prism Version 7.

Small molecular enzyme inhibitors, often based on the molecular structure of the native substrate of the enzyme have proved promising in rational drug design, with bacterial enzymes deemed effective targets for therapeutic intervention in human bacterial infections^21^. Enzymes associated with *P. mirabilis* virulence have previously been targeted as therapeutic options. Carson et al*.,* previously described the development and analysis of a library of N-alpha mercaptoamide dipeptide inhibitors against Zap A protease (a virulence factor known to play a key role in *P. mirabilis* pathogenesis)^22^. Despite their in vitro success in inhibiting ZapA, these inhibitors showed negligible activity against urease (unpublished data, not shown). Therefore, a design is proposed whereby the bulky, peptide-containing regions of the inhibitor are eliminated, leaving only the thiol-containing amide ‘warhead’ of the molecule, 2-mercaptoacetamide (2-MA) (Fig. 1a). The conserved thiol moiety of the inhibitor shows structural potential for the inhibition of urease. Since there are structural similarities with urea, there is potential to allow for a binding mode that resembles that of the native enzyme–substrate complex and the consideration of the innate substrate specificity of the urease enzyme. Furthermore, the proposed 2-MA inhibitor contains a thiol moiety, which is known to display active inhibition of the urease enzyme, as described elsewhere^23,24^. Urease inhibitors have been extensively examined by previous researchers as described by^25,26^. However, many focus on their use in agriculture and few test their use on medicinally relevant bacterial ureases such as those from *P. mirabilis*.

Here, we describe the preparation and in vitro evaluation of 2-MA as a small molecular urease inhibitor, which can prevent encrustation and blockage of urinary catheters in a physiologically representative in vitro model of the catheterized urinary tract. Computational docking experiments identify 2-MA as a competitive inhibitor against urease and shows the possibility for rational design of future inhibitory candidates. Furthermore, quantum mechanical (QM) computational modelling hypothesizes two possible 2-MA’s binding mechanisms and allows comparison to the licensed drug AHA.

## Results and discussion

This study aimed to identify an inhibitor of the urease enzyme, which is produced by the uropathogenic bacteria *P. mirabilis*. Inhibition of this target was intended to disarm *P. mirabilis*’s ability to form crystalline biofilms, thus preventing encrustation and blockage of long-term urinary catheters.

### Enzyme kinetics

Urease from *Canavalia ensiformis* (*C. ensiformis*) was used as a model urease to determine the kinetics and analyze the initial inhibitory affinity of 2-MA. The active site of urease is conserved between *C. ensiformis* and *P. mirabilis* urease (Supplementary Fig. 3). Michaelis–Menten analysis of enzyme kinetics showed that a sigmoidal curve fit agreed with the observed kinetic data (R^2^ = 0.9959) compared with a hyperbolic curve fit (R^2^ = 0.9537) (Fig. 1b,c).

The V_max_ was calculated as 1.31 × 10^–2^ M and the K_M_ as 0.16 M (Fig. 1c). The K_M_, 0.16 M, was compared to literature values of 2.7–8.81 mM^27^. A Hill coefficient of 2.19 suggest cooperative binding of urea to urease. This agrees with literature on the mechanism of urease where both a nucleophilic water molecule and urea bind to the bi-nickel active site, this was further confirmed through computational analysis^28^. Analysis of Michaelis–Menten parameters in the presence and absence of 2-MA indicates the type of inhibition (Supplementary Table 1). The V_max_ remains approximately the same upon addition of 2-MA (within the error margins), whilst K_M_ increases suggesting decreased affinity of urea for the active site urease, indicating that 2-MA is binding competitively and thus is competing directly with the substrate for the active site (Supplementary Table 1). It is worth noting, however, that there are limitations to the Michaelis–Menten model. Whilst it has been used widely for over a century to estimate enzyme kinetic parameters from substrate reaction curves, this canonical approach only works in limited conditions (such as when there is a large excess of substrate over enzyme). Even when this condition is satisfied, the identifiability of parameters is not always guaranteed, and often not verifiable in practice^29^. Furthermore, molecular docking experiments suggested that 2-MA was binding within the active site and thus is competitively inhibiting urease. The mechanism of enzyme inhibition, as well as the inhibitor constant (K_i_) were confirmed via the linear graphical method known as the Dixon plot. The effect on the enzymatic initial rate (V_0_) is determined at two or more substrate concentrations over a range of inhibitor concentrations. For competitive inhibition, a plot of 1/initial rate vs. inhibitor concentration will yield a series of straight lines that intersect above the X-axis; the abscissa at which they converge represents the − K_i_^30^. This provides a quantitative measure of inhibitory potency that is independent of substrate concentration, and provides information about the binding affinity of an inhibitor^31^. Using the Dixon method, it was seen that 2-MA competitively binds to urease (Fig. 1d). The K_i_ was calculated as 2.95 mM, the K_i_ of AHA for urease has been previously described as 5.7 ± 0.4 μM, and 0.053 mM, for *C. ensiformis* and *P. mirabilis* urease, respectively^32,33^. Therefore, 2-MA is less potent than AHA however, AHA causes severe toxicity to the patient and 2-MA efficacy in preventing blockage of catheters also needs to be examined. Half-maximal inhibitory concentration (IC_50_) is the inhibitor concentration at which the activity of the enzyme is reduced by 50%, when compared to the activity of the enzyme in the absence of the inhibitor. Determination of IC_50_ of a compound provides a further measure of the antagonistic potency, in a manner independent of defined experimental conditions (such as substrate concentration). Dose–response curves and subsequent determination of IC_50_ for both 2-MA and AHA are shown in Fig. 1e,f. The calculated IC_50_ for 2-MA (57.9 mM) was approximately tenfold lower than that of AHA (4.8 mM). The IC_50_ indicated significantly lower potency for 2-MA compared to the currently licensed therapeutic AHA. Nevertheless, since determination of the IC_50_ yields no pharmacokinetic information, or data regarding biocompatibility and cytotoxicity, further biological evaluation was required to allow conclusions on the suitability of 2-MA as a drug candidate to be drawn.

### In silico modelling of urease-2-MA interaction

**Figure 2:**
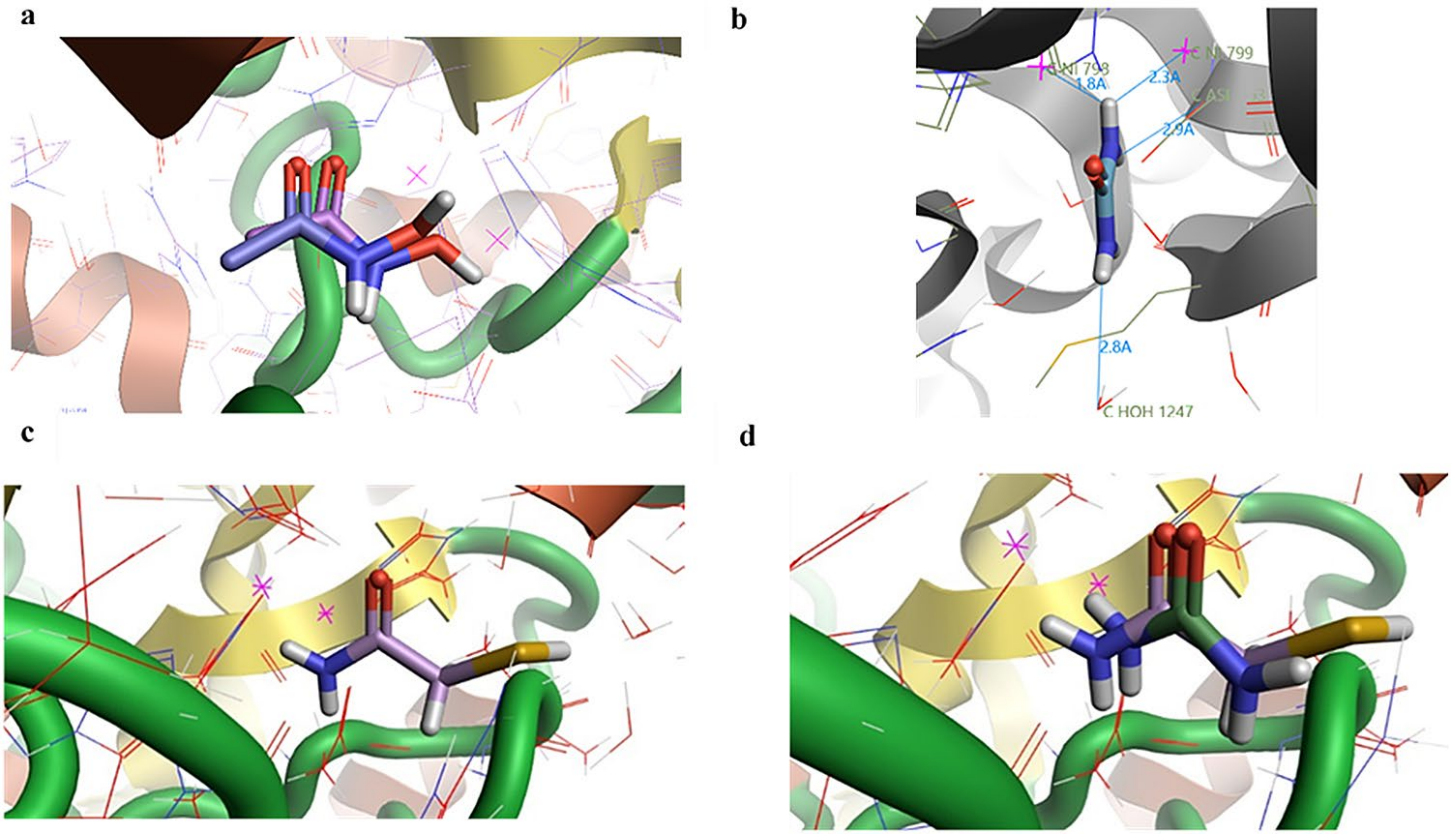
(**a**) AHA self-docked into the active site of urease (from *S. pasteurii*, PDB = 4UBP^31^). The bottom compound is crystallized AHA overlaid with the docked AHA. A RMSQ of 0.387 Å is calculated for the difference between the crystallized AHA and the docked AHA. (**b**) Analysis of the contacts of docked urea with the active site of urease. Urea is coordinating with the bi-nickel active site (Ni shown as pink crosses) and with D363, as well as interacting with surrounding water molecules. Distances are marked with blue lines and measured in Å. (**c**) 2-MA docked to urease, 2-MA binds to H222, H275, and D363. (**d**) Overlay of 2-MA docking and urea docking to the active site of urease. Docking carried out using Flare (Flare 3.0.0, Revision 38710; Cresset, Litlington, Cambridgeshire, UK).

To further understand the mechanism of inhibition and confirm that 2-MA was binding to the active site of urease, computational docking of 2-MA onto the crystal structure of urease was carried out. Crystal structures of *P. mirabilis* urease are not available at time of writing, however, the active site is well conserved (Supplementary Fig. 3). The crystal structure of urease (protein data bank (PDB): 4UBP) from *Sporosarcina pasteurii* (*S. pasteurii*) (formally *Bacillus pasteurii*) was chosen owing to its co-crystallization with AHA and high crystal resolution (1.55 Å), thus allowing accurate determination of bond distances in docked compounds^34^. We performed ‘self-docking’, whereby crystallized AHA was used as a control to check the computational docking before docking the 2-MA. Figure 2a indicates that docked AHA reported good confirmation to the crystalized AHA, with a root mean squared deviation (RMSD) of 0.387 Å, where an RMSD of < 2 Å is considered a ‘well-docked’ ligand^35^. It was concluded that the docking software was performing as expected and would provide realistic docking for 2-MA. Compounds were molecularly docked into the active site of urease and their contacts with the protein examined. For protein–ligand interactions, hydrogen bonding as well as hydrophilic-hydrophobic interactions are important for drug binding. Owing to the metallocentre of urease, Ni ions can also be involved in coordinating ligands, such as that observed in thiol inhibitors^36^. By examining the distances between the compound and the protein, hydrogen bonding can be predicted, most hydrogen bonds have a bond distance between 2.7 and 3.1 Å^37^.

Urea was docked into the active site, it coordinates with Ni ions, with bonding distances calculated to be 1.8 Å to Ni(1), and 2.3 Å to Ni(2) (Fig. 2b). Urea also binds to D363 at a distance of 2.9 Å, as well as a water molecule at 2.8 Å. The binding contacts of urea with the active site of urease, agreed with the mechanism of urease proposed by Amtul et al*.*^28^. Figure 2c shows docked 2-MA. This is in agreement with the Dixon Plot and Michaelis–Menten parameters in the presence of 2-MA, to confirm that 2-MA is inhibiting urease by competitive inhibition (Fig. 1d, Supplementary Table 1). The carbonyl group appears to bind with amine from H222, with a bond distance of 1.9 Å. The amine from 2-MA forms hydrogen bonds with H275 with a bond distance of 2.6 Å, and D363 with a bond distance of 1.9 Å. The sulfur forms hydrogen bonds with two water molecules found in the active site, upon comparison to the crystallized structure of urease with β-mercaptoethanol, it is observed that the sulfur is not bound with the Ni ions as observed in the crystallized structure^38^. It could be hypothesized, that the amide group is more favorable to chelating the Ni ions compared to the sulfur as it is a substrate mimic. However, crystallography studies are required to further confirm this 2-MA binding behavior. It is hypothesized that the potency of 2-MA could be improved by chain extension from the methylene group to the develop contacts with the surrounding amino acids. Furthermore, 2-MA conserves the amide functional group from urea therefore, superposition of urea and 2-MA determined that both compounds dock in the same orientation (Fig. 2d). Both the carbonyl and the amide group of urea and 2-MA coordinate to the Ni ions from urease. The docking score uses mathematical methods to predict compounds docking pose to a receptor^39^. Whilst the docking score alone does not provide an accurate model of binding, when combined with successful self-docking data, it can be used successfully to analyze compound analogues^40^. The docking scores for urea, 2-MA, and AHA were − 3.814, − 6.347, and − 6.534, respectively. The docking score for urea is higher than 2-MA and AHA, suggesting that the compounds are binding better than the natural substrate. This is likely due to the higher amount of contacts 2-MA and AHA are making within the active site. 2-MA and AHA have very similar docking scores indicating a similar binding strength to the active site. However, AHA has a lower score than 2-MA, thus the docking scores agree with the kinetic data, suggesting that AHA has a higher affinity than 2-MA for urease’s active site.

### QM modelling of *S. pasteurii* urease active site

An inherent limitation with a classical molecular docking approach is that one cannot account for QM effects such as bond breaking and formation, nor observe directional binding to metal centers, which arise as a direct result of orbital overlap. To this end, further QM computational studies were undertaken to supplement the docking analysis.

**Figure 3:**
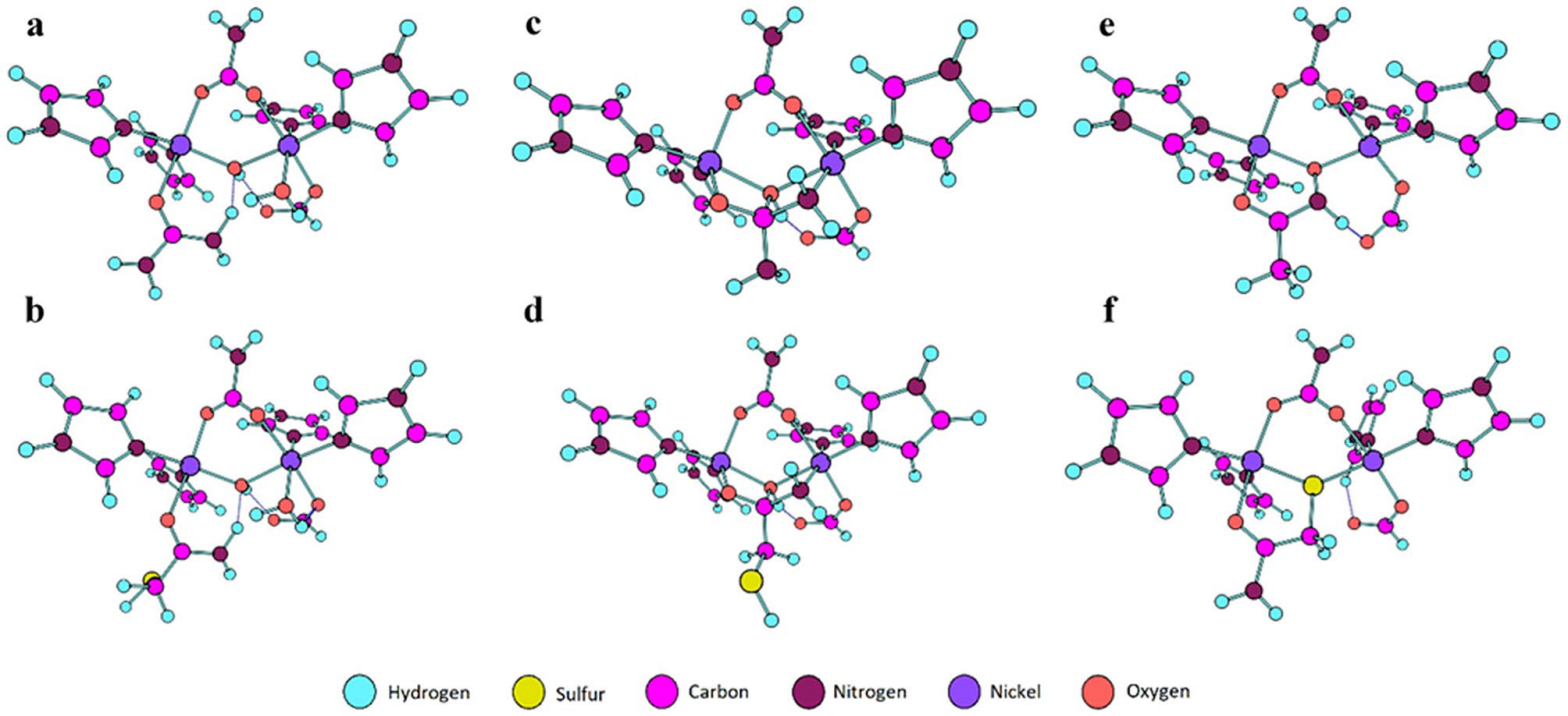
PM6 optimized structures of the active site of *S. pasteurii* urease. (**a**) monodentate urea binding as shown by Nordlander et al.^41^. (**b**) 2-MA mimicking monodentate binding of urea. (**c**) Binding of urea through a tetrahedral intermediate as shown by Mazzei et al.^42^. (**d**) 2-MA bound through a tetrahedral intermediate. (**e**) AHA anion binding in same fashion as found in crystal structure (PDB = 4UBP). (**f**) 2-MA anion mimicking the binding of the AHA anion. Graphics generated using Chemcraft^43^.

Structures were selected based on molecular docking configurations and crystal structure data. These structures were then optimized to potential energy minima. Figure 3 shows the optimized structures of the active site of *S. pasteurii* urease complexed with urea (Fig. 3a,c), neutral 2-MA (Fig. 3b,d), the AHA anion (Fig. 3e) and the 2-MA anion (Fig. 3f).

Potential energies for these structures can be found in Table 1. It must be noted that the energies presented in this report are enthalpies of binding, as opposed to free energies, upon which true binding will depend. It is therefore important that enthalpies are compared for binding events where the entropy change is likely to be similar, such as for a binding process which liberates the same number of small molecules (*i.e.* water). When this is not the case, one must be aware that enthalpy alone, whilst a good indicator for binding affinity, does not provide the full picture that the free energy of binding would. The binding enthalpy of urea in Fig. 3a is − 77.7 kJ mol^–1^, which is similar to the binding enthalpy of monodentate bound 2-MA in Fig. 3b, − 64.6 kJ mol^–1^. In this mode of binding, urea is predicted to be a better ligand than 2-MA by roughly 13 kJ mol^–1^. This suggests that this is not the binding mode that 2-MA adopts in the active site, as it is energetically favorable for urea to displace 2-MA in this mode. Attempts to optimize cross chelating structures of urea and 2-MA (Ni(1)–O=C and Ni(2)–NH_2_), in a fashion similar to that shown by the substrate analogue, boric acid^44^, led to attack by the bridging hydroxide on the electrophilic carbonyl. This concerted cross coordination and nucleophilic attack mechanism is supported by previous observations and calculations made by Musiani et al*.* on the mechanism of urease catalysis^45^. This nucleophilic attack by the bridging hydroxide leads, in both cases, to a tetrahedral intermediate, as seen in Fig. 3c,d. When bound in this fashion the 2-MA ligand is 28 kJ mol^–1^ more stable than urea. Reaction via a tetrahedral intermediate as the mechanism for urease has also been supported by recent X-ray crystallographic data on the urease-urea complex by Mazzei et al*.*^42,46^. The difference in binding energies suggest that in this binding mode, 2-MA is favored over urea and would lead to inhibition of enzyme function. Structures in Fig. 3e,f are the optimized structures of the AHA anion bound to the urease active site, as determined from crystallographic data, and the 2-MA anion mimicking this binding mode^34^. Comparison of the binding energies of these structures shows that this mode of binding is highly unfavorable for 2-MA. Noticeably, the binding enthalpy for AHA in Fig. 3e is calculated as 70 kJ mol^–1^ higher than that of urea in Fig. 3a. Whilst this suggests that urea binding in Fig. 3a is enthalpically favorable when compared to AHA binding, when compared to urea binding in a tetrahedral intermediate (Fig. 3c), which is believed to be an intermediate along the urease mechanistic pathway, AHA has a favorable binding enthalpy of approximately 30 kJ mol^–1^. Both in silico studies showed that 2-MA, when bound as a tetrahedral intermediate, and AHA were found to bind preferably to the active site when compared to urea. This indicates the potential of 2-MA as a drug candidate/template for further drug development, this was determined by the docking scores and binding energies. Further QM studies are ongoing, with the aim to incorporate the effect of the protein environment on ligand binding and predict binding enthalpies for 2-MA analogues in the tetrahedral binding mode.

**Table 1:** Potential and binding energies of structures Fig. 3a–f.

	Ligand	Waters molecules exchanged^a^	Potential energy (Hartrees)^b^	Binding energy (kJ/mol)^c^
a	Urea	1	− 3140.999223	− 77.73342713
b	2-MA	1	− 3140.994208	− 64.56565459
c	Urea	2	− 3064.545142	22.46070774
d	2-MA	2	− 3064.555789	− 5.491864421
e	AHA	3	− 2988.140894	− 8.180953242
f	2-MA	3	− 2988.079474	153.0776375

^a^The number of water molecules exchanged with the ligand upon binding to the wild type active site.

^b^Potential energies are quoted for the unrestricted Becke, 3-parameter, Lee–Yang–Parr (B3LYP)^47–50^ functional and 6-31G*/SDD basis sets^51,52^ (UB3LYP/6-31G*/SDD).

^c^Binding energies were calculated as potential energy of the structure plus the potential energies of any liberated water molecules (aqueous solvation with PCM, ε = 80), minus the potential energies of the wild type active site and aqueous solvated ligand (PCM, ε = 80). All calculated energies can be found in Supplementary Table 2.

### Investigating 2-MA inhibition on *P. mirabilis* urease

Evaluation of the functionality of 2-MA against urease from *P. mirabilis* is crucial for understanding whether 2-MA is effective in a clinical scenario. Urease is a intracellular enzyme, thus 2-MA must be able to cross the bacterial membrane^6,53,54^. Analysis of the growth of *P. mirabilis* (strain B4) in the presence of 2-MA and AHA allows the maximum tolerable concentration of the drug to be determined, by comparison to the B4 control (untreated control). The maximum tolerable concentration is defined as the concentration of the drug at which the growth of *P. mirabilis* remains unaffected. By maintaining bacterial cell viability, evolutionary selection pressures are not introduced, thus the likelihood of resistance occurring decreases^21,55^. It was determined using a standard microbroth dilution method that the maximum tolerable concentration for both 2-MA and AHA is 10 mM (Supplementary Fig. 4). For in vitro whole-cell urease evaluation, it was imperative to employ a concentration of drug that does not affect bacterial cell viability, such that any reduction in urinary crystal formation may be attributed solely to urease inhibition, not a reduction in bacterial bioburden. Therefore, the selected dosage for all further biological evaluation was 10 mM for both 2-MA and AHA.

**Figure 4:**
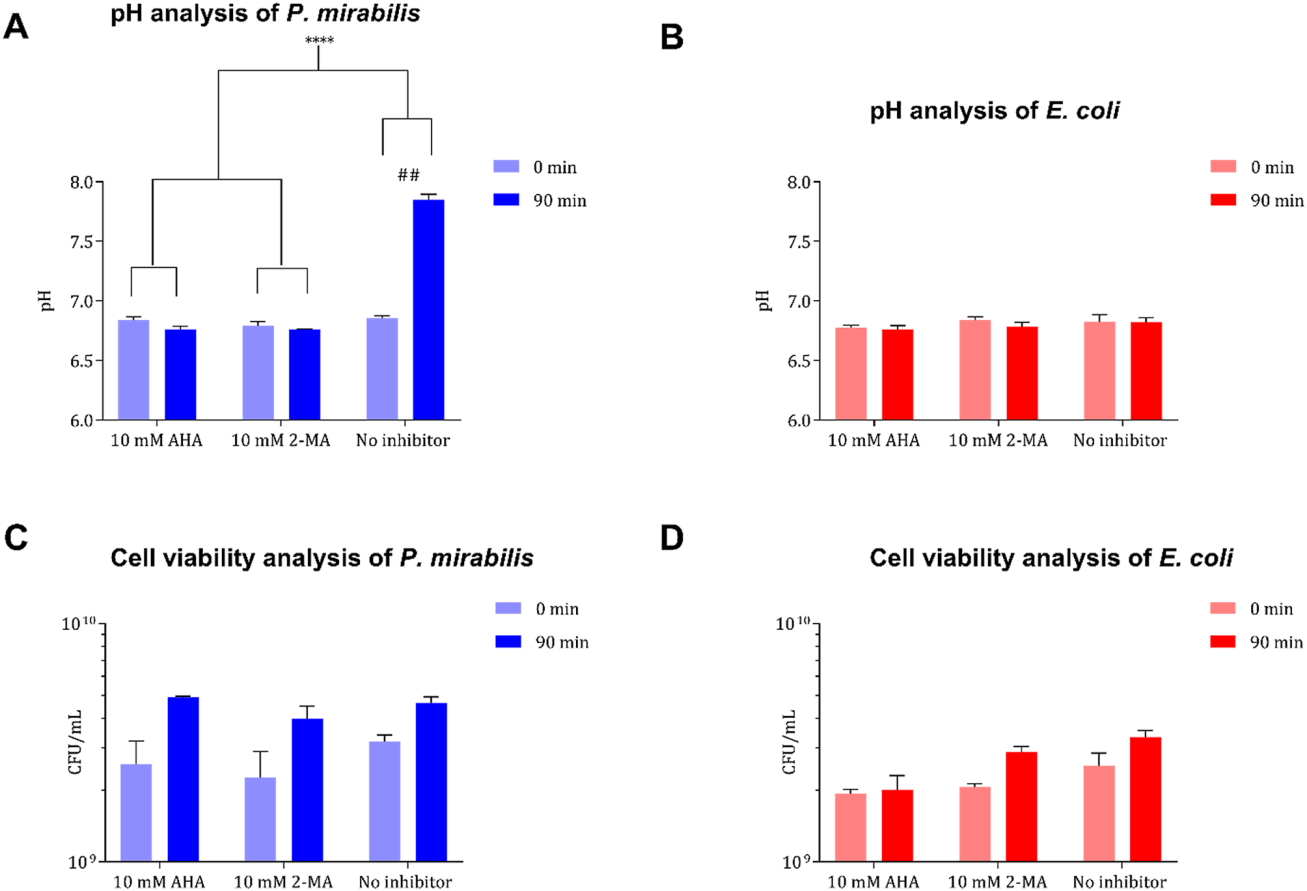
Whole cell evaluation of 2-MA and AHA. (**A**, **B**) Ability to penetrate bacterial cell membrane and inhibit intracellular urease activity was determined via measurement of the macromolecular changes in pH. (**C**, **D**) Viable cell counts were performed to ensure the viability of cultures before (0 min) and after (90 min) treatment with 10 mM dosage of inhibitory drugs. Evaluation was performed on both urease-positive (*P. mirabilis* B4) (blue), and urease-negative (*E. coli* NSM59) (red) uropathogenic clinical isolates. Data shown are the mean of three biological replicates, error bars represent SEM ****p < 0.0001, ^##^*p* < 0.005.

Penetration through the cell membrane provides a significant challenge in the design of small molecular drugs such as enzyme inhibitors. Intracellular uptake is frequently confirmed via measurements of a biological effect generated only when the payload is successfully delivered to the cytoplasm. Macromolecular changes in pH, over a small time period, were used to assess whether 2-MA was passing through the membrane of *P. mirabilis* (Fig. 4). Changes in pH caused from the production and accumulation of ammonia in the urine were used as a proxy indicator of enzyme activity. Comparison to a uropathogenic strain of *Escherichia coli* (*E. coli*), a urease-negative control, indicates that the change in pH is as a result of urease activity from *P. mirabilis*. The measured pH (after 90-min incubation) between *P. mirabilis* and *E. coli* was found to be significantly different (*p* = 0.0001) (Fig. 4A,B). Exposure of *P. mirabilis* to 10 mM 2-MA prevented urinary pH elevation compared to an untreated control (*p* < 0.001) (Fig. 4A). Urease inhibition in the presence of 2-MA (10 mM) was comparable to AHA (10 mM), both compounds exhibited significant enzyme inhibition. There was no alteration in urinary pH for cultures of urease-negative *E. coli* (Fig. 4B). Furthermore, there is no significant difference in the bacterial viability between *P. mirabilis* and *E. coli* at the start and the end of the experiment for either of the drugs tested (Fig. 4C,D).

### In vitro bladder model testing

**Figure 5:**
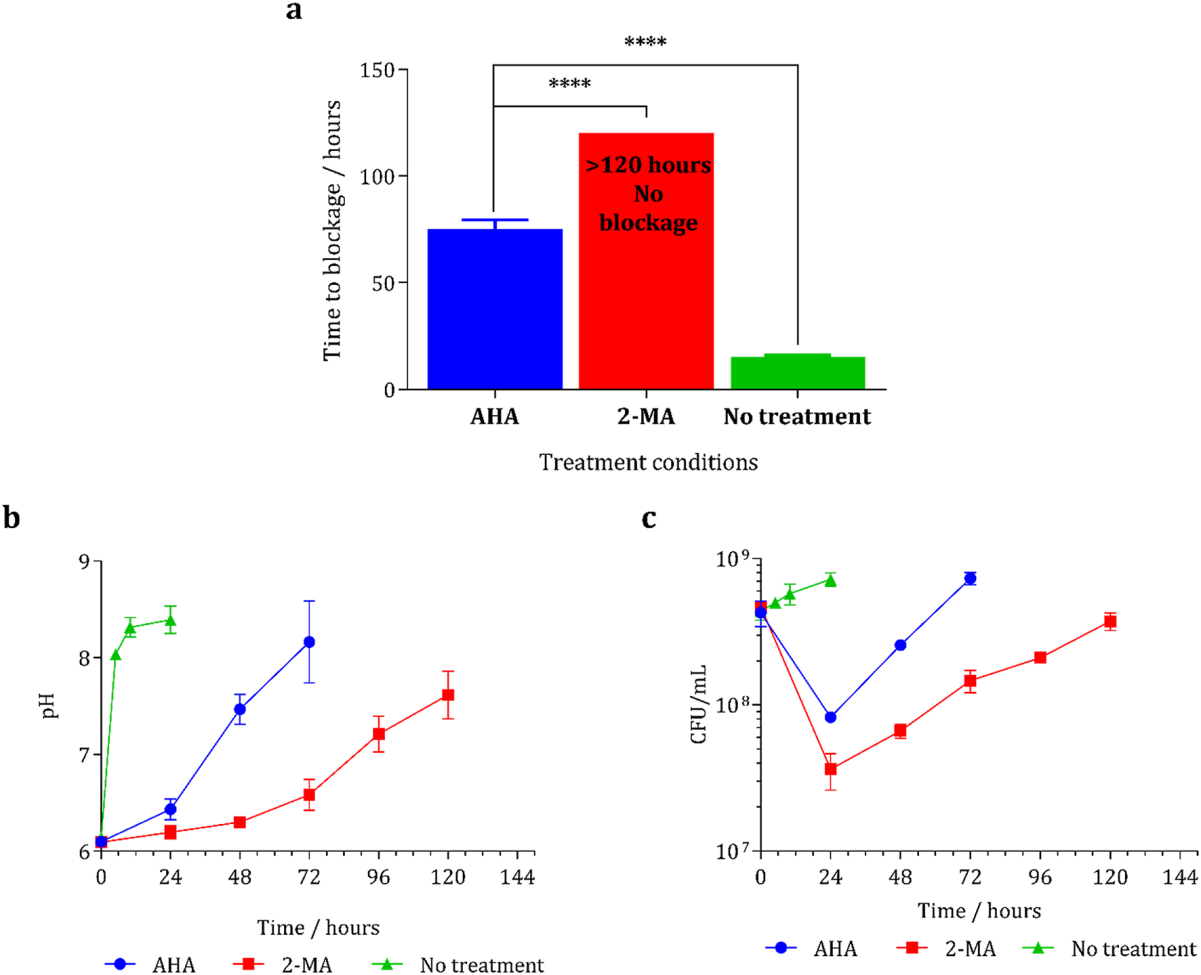
(**a**) measuring the time to block of in vitro bladder models comparing *P. mirabilis* infection with 2-MA, AHA or no treatment (control). Analysis of the in vitro bladder models infected with *P. mirabilis* and treated with either 2-MA, or AHA, or no treatment. (**b**) pH measurements of the residual urine. (**c**) measurements of the bacterial biomass. Results are from three biological replicates, error bars represent SEM, ****p < 0.0001.

The efficacy of 2-MA in a clinically representative system was assessed using in vitro bladder models, as previously described^56^. Physiological conditions (including temperature and urine flow rate) were maintained within the bladder models, which were inoculated with *P. mirabilis* and *E. coli* at 10^8^ colony forming units (CFU)/mL to model a late stage CAUTI. 2-MA and AHA drugs were added by direct dissolution into the urine reservoirs at 10 mM concentration. The blockage of urinary catheters and subsequent termination of urine flow was defined as the experimental end point (Fig. 5a). The performance of 2-MA was compared directly to that of AHA, in order to reference the extension of catheter lifetime under the same experimental conditions. Periodic measurements of bladder conditions (pH and CFU/mL) were performed throughout the experiment, to monitor the urease activity as a function of residual urine pH, as well as the viability of colonizing bacteria (Fig. 5b,c). Both inhibitors achieved significant extension of catheter lifetime compared to the untreated control. When urease activity was uninhibited, blockage of control models occurred after 15 h. Treatment with AHA extended average blockage time fivefold, blockage was visually observed at 74 h (*p* < 0.0001). Surprisingly, treatment of *P. mirabilis*-infected models with 2-MA further extended catheter lifetime within the in vitro model, such that blockage was not observed throughout the 5-day duration of the experiment. Reservoirs of artificial urine media were exhausted 120 h after model start, at which point catheters treated with 2-MA were still draining freely (Fig. 5a). This in vitro model shows the ability of 2-MA to significantly outperform AHA, the only currently marketed urease inhibitor. 2-MA successfully prevented blockage of the catheters and significantly extended the lifetime of the catheter by an extra 46 h compared to AHA.

Within the untreated control models, the pH of artificial urine media within the bladder rapidly increased, owing to the urease-mediated urea hydrolysis within the *P. mirabilis* population. The pH of residual media was elevated to pH 8 within 5 h of the experiment start. This was accompanied by an increase in viable bacterial cells within the bladder, since urease expression was able to facilitate rapid colonization and biofilm formation. Local supersaturation and precipitation of crystalline materials within the catheter lumen and eyehole region therefore elicited total occlusion of the catheter within 15 h. In contrast, pH elevation within treated models was unable to surpass to nucleation point (≈ pH 7.4) until approximately 45 and 108 h for AHA and 2-MA, respectively. Eventual elevation of urine to pH 8 in AHA-treated models was accompanied by catheter blockage by crystalline biofilm formation. Models treated with 2-MA, were unable to reach pH 8 within the experimental term, thus the degree of crystalline biofilm formation was insufficient to result in catheter blockage (Fig. 5b). Both the AHA and 2-MA-treated models underwent an approximately 1-log reduction in viable cell count within the first 24 h of the experiment. *P. mirabilis* virulence factors (particularly urease) are known to play an extensive role in bacterial pathogenesis and biofilm formation^4^. It is therefore unsurprising that inhibition of urease activity manifested as a decrease in bacterial colonization. It has been shown that urease-negative mutants, colonize the urinary tract 100-fold less efficiently than the parent strain^57,58^. It is likely that the reduction in adherence resulted in direct elution of a large portion of the bacterial population from the bladder in the early stages of infection (Fig. 5c). Disparities in the degree of encrustation on catheter luminal surfaces at time of AHA-treated model blockage (74 h) and unblocked 2-MA-treated model (120 h) were observed visually. Total occlusion of the eyehole and drainage lumen were observed in the catheter removed from the blocked AHA treated model (Supplementary Fig. 5a). Significantly less crystalline material was observed within the catheter removed from the 2-MA treated model (Supplementary Fig. 5b).

**Figure 6:**
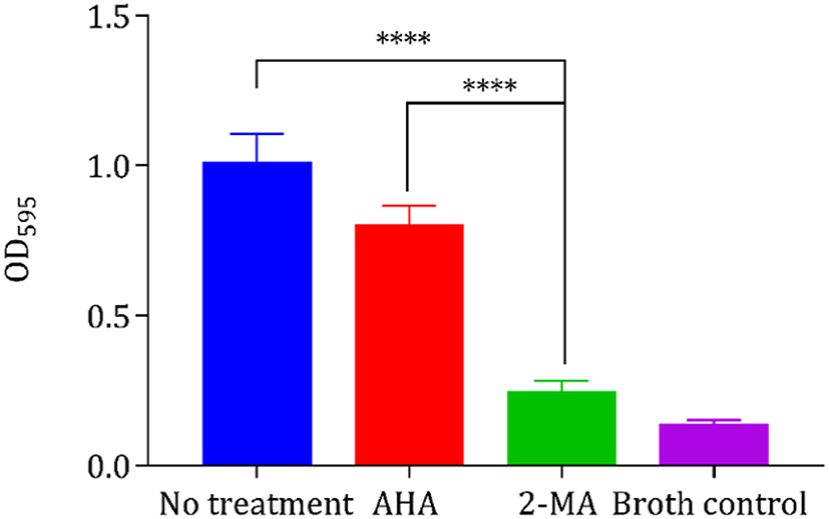
Quantitative measurement of *P. mirabilis* B4 static biofilm inhibition by 10 mM 2-MA and AHA. Quantification was performed via crystal violet biofilm staining. Results are from three biological replicates. Error bars represent SEM. ****p < 0.0001.

In order to further elucidate why 2-MA has outperformed AHA despite having a lower potency in initial IC_50_ studies, the ability of 2-MA to inhibit the formation of *P. mirabilis* biofilms was investigated using a simple in vitro crystal violet staining model. Since treatment of the in vitro catheterized bladder with 2-MA resulted in significant elution of planktonic cells from the bladder in the early stages of the experiment, it was hypothesized that this drug is preventing the early colonization of a biofilm on the catheter’s luminal surfaces. The ability of both enzyme inhibitory drugs to prevent biofilm formation of *P. mirabilis* is shown in Fig. 6. At the therapeutic concentration of 10 mM, 2-MA showed significantly greater ability to prevent formation of biofilms than AHA (*p* < 0.0001), although it did not completely prevent formation (*p* = 0.0214).

Since previous investigation into the antimicrobial susceptibility of *P. mirabilis* to both 2-MA and AHA has confirmed that 10 mM concentrations are unable to induce bacterial cell death (Supplementary Fig. 4), it appeared that the reduction in biofilm formation (and hence extension of catheter lifetime) is owing to the prevention of catheter colonization and adherence to the catheter surface.

### Cytotoxicity testing

**Figure 7:**
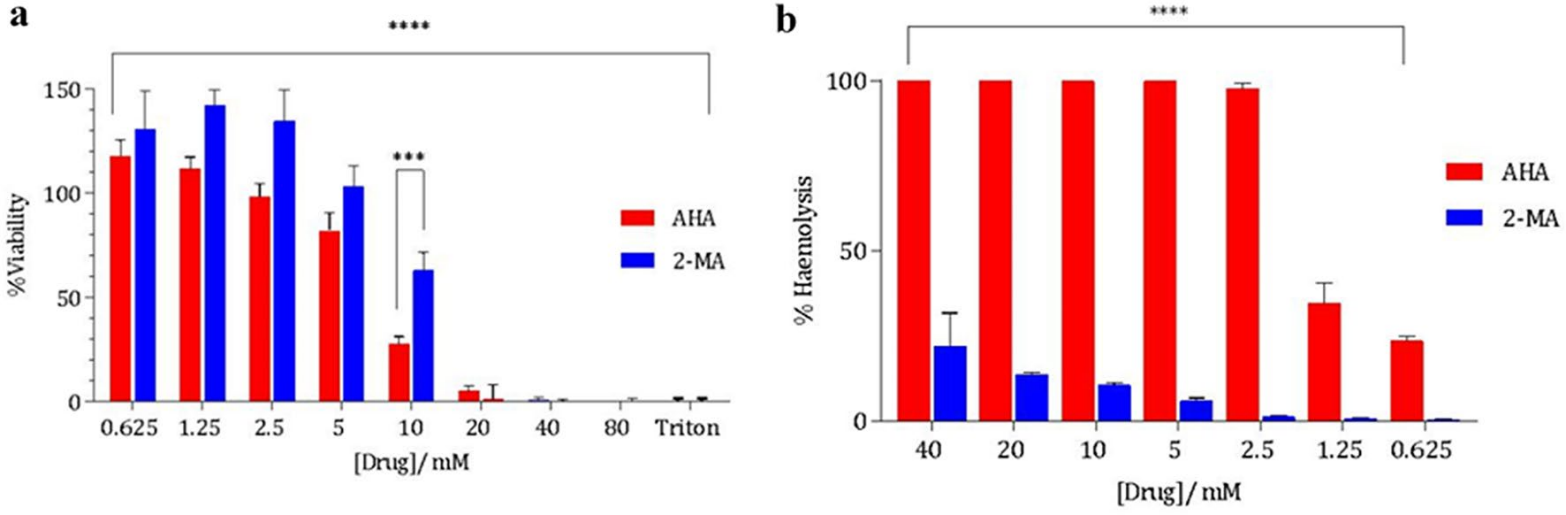
(**a**) CyQUANT XTT assay measuring the viability of HaCaT keratinocytes at varying concentrations of 2-MA and AHA. Corrected against absorbance of 450 nm of untreated control to give % viability. At 10 mM there is a significant difference between the viability observed to treated cells with AHA and 2-MA. Triton X-100 was used as positive control. (**b**) Percentage ex vivo hemolytic activity of dose-dependent 2-MA and AHA treatment human erythrocytes. Drug concentrations 0.625–40 mM. Hemolysis was calculated by correcting to the positive lysis control Triton X-100. Results are from three biological replicates. Error bars represent SEM. ****p < 0.0001, ***p < 0.001.

Within cell culture systems, a compound is considered cytotoxic if it interferes with cellular attachment, significantly alters morphology, adversely affects growth rate, or causes cell death^59^. Cytotoxicity can be measured by measuring cellular metabolism, 2-MA was directly compared to that of AHA using a HaCaT immortalized keratinocyte cell line using the XTT cell metabolism assay. 2-MA consistently showed significantly less cytotoxicity than AHA. At 10 mM, which has been used to treat the *P. mirabilis*, reduced viability is observed for both AHA and 2-MA. Investigating the cytotoxicity against HaCaT cells indicated that, as previously observed by Williams et al., AHA was toxic towards mammalian cells (Fig. 7a)^18^. Incubation of cells with 10 mM 2-MA resulted in 60% cell viability, whereas under the same conditions AHA resulted in a decrease to 30% viability. Any reduction in viability of HaCaT cells is discouraging in drug development however, 2-MA offers a good starting point for further optimization. The cytotoxicity of AHA agrees with the literature and has also been reported to behave synergistically with bacterial virulence factors leading to increased toxicity^60^.

The degree of hemolytic anemia induced by a drug may be evaluated ex vivo by assessing the dose-dependent liberation of hemoglobin from human red blood cells via the measurement of absorbance at 415 nm. Obtained values were corrected against media controls, and degree of hemolysis expressed as a percentage of the positive lysis control (Triton X-100). Percentage hemolysis for both 2-MA and AHA (both dissolved in phosphate-buffer saline (PBS)) at varying concentrations is shown in Fig. 7b. Toxic hemolytic activity was significantly lower for 2-MA than AHA (*p* < 0.0001). Of particular relevance was the result at the defined therapeutic concentration (10 mM), where % hemolysis was observed to be 10.7% ± 0.8 for 2-MA; an 89% reduction from the value observed for AHA at the equivalent concentration. Even at the highest evaluated concentration of 40 mM, observed hemolysis was still 78% lower following treatment with 2-MA in comparison to AHA (*p* < 0.0001 for all comparative concentrations) (Supplementary Fig. 6).

## Conclusions

Drugs that function as enzyme inhibitors constitute a significant portion of the orally bioavailable therapeutic agents that are in clinical use today. The ‘disarming’ of potent bacterial virulence factors without placing a selective pressure on the cells themselves comprises a powerful therapeutic approach in light of the dawn of the post-antibiotic era. Kinetic and in silico analysis indicates that 2-MA is a competitive inhibitor against urease. 2-MA was shown to out-perform AHA during in vitro bladder model evaluation and cytotoxicity studies. QM computational analysis identified two possible competitive binding mechanisms for 2-MA: either mimicking monodentate urea binding or binding as a tetrahedral intermediate. Of these two modes, the tetrahedral intermediate is likely to contribute to greater inhibition of urease action based on our calculations. The licensed inhibitor, AHA, has a higher affinity for urease, however this comes at a significant cytotoxic cost, both to bacterial and mammalian cells^17,19^. The trade-off between toxicity and potency is common in drug discovery; multi-optimization objective methods have been developed to rank compounds that fully agree with multiple criteria^61^. This is in contrast to traditional methods where objects are tackled sequentially, normally starting with potency^62^. We have analyzed 2-MA for multiple criteria, including potency and toxicity as well as examining its binding mechanism. The relatively low toxicity, low cost, and efficacy of 2-MA not only in inhibiting urease but also, in inhibiting biofilm formation suggest that 2-MA may have clinical utility in preventing urinary catheter blockage. Although the working concentration of 10 mM 2-MA used in this study is high, direct bladder washouts using a three-way Foley catheter, means that 2-MA could be introduced as a weekly prophylaxis in the clinic, replacing AHA. 2-MA additionally offers a suitable platform from which more potent analogues may be developed through the use of rational drug design.

## Methods

### General remarks

Solvents and reagents were purchased from commercial suppliers and used without further purification unless otherwise stated. ^1^H NMR was recorded on a Bruker 400 MHz. Chemical shifts (δ) are reported in ppm, and referenced to residual solvent peaks. Splitting patterns are defined as singlet (s), doublet (d), triplet (t), quartet (q), multiplet (m) or broad (br) where appropriate. The HPLC-ESI-TOF analysis was conducted using an electrospray time-of-flight (MicrOTOF) mass spectrometer (Bruker Daltonik GmbH, Bremen, Germany), which was coupled to an Agilent HPLC stack (Agilent, Santa Clara, CA, United States) consisting of Agilent G1312A binary pump with G1329A autosampler and G1316A column oven. Analyses were performed in ESI positive mode with capillary voltage at 4500 V, nebulizing gas at 2.2 bar, drying gas at 10.2 L/min at 220 °C. The TOF scan range was from 50 to 750 mass-to-charge ratio (m/z). The system was configured with a switching valve to perform flow injections, where 10 µL injections were made. Mobile phases A and B consisted of H_2_O and MeOH at 0.4 mL/min, respectively. The TOF was calibrated with a 10 µL sodium formate calibrant solution injection prior to the flow injection run. The calibrant solution consisted of three parts of 1 M NaOH to 97 parts of 50:50 water:isopropanol with 2% formic acid. Automated data processing was performed using the Compass Data Analysis software scripts (Bruker Daltonik GmbH, Bremen, Germany). PBS tablets were purchased from Fisher Scientific, sodium chloride (137 mM), phosphate buffer (10 mM), and potassium chloride (2.7 mM), pH 7.4.

#### 2-MA synthesis

2-MA was prepared according to Latli et al.^63^. Briefly, methyl thioglycolate (3.60 g, 34 mmol, (Merck, Germany)) was stirred for 14 h under a nitrogen atmosphere, at room temperature (RT), with methanolic ammonia (7 M, 45 mL (Merck, Germany)). The solvent was removed in vacuo producing 3.1 g of white solid (98%). Confirmation of purity was carried out using ^1^H NMR (400 MHz, CDCl3) δ: 1.97 (s, 1H, SH), 3.22 (s, 2H, CH_2_), 6.38 (br, 1H, NH_2_), 6.62 (br, 1H, NH_2_) in agreement with previously published spectra Bockman et al. (Supplementary Fig. 7)^64^. Additionally 2-MA was analyzed by HPLC-ESI-TOF, C_2_H_5_NOS was detected as [M + Na] + ion with − 0.60 ppm mass error. Theoretical [M + Na] + 113.9984 m/z, observed 113.9983 m/z.

#### Preparation of *P. mirabilis, E. coli* and quantification

Uropathogenic bacteria, *P. mirabilis B4* and *E. coli* NSM59 were from the laboratory’s collection. *E. coli* and *P. mirabilis* were cultured from 15% (v/v) glycerol on solid state agar plates. *E. coli* used Luria–Bertani (LB) agar plates, incubated statically at 37 °C overnight. Due to *P. mirabilis*’ swarming ability, to achieve single colonies, non-swarming LB (NSLB) composed of tryptone (10 g/L (Merck, Germany)), yeast extract (5 g/L (Merck, Germany)) and bacteriological agar (15 g/L (Merck, Germany)) is required. *P. mirabilis* NSLB plates were incubated the same as *E. coli*. Single colonies were picked from the plates and grown in 10 mL of LB broth overnight at 37 °C with shaking (200 rpm). Quantification is carried out using the Miles, Misra and Irwin method^65^. Plates are incubated at 37 °C statically overnight. CFU/mL were counted

$$\text{CFU}/\text{mL }= \frac{\text{average number of colonies}}{\text{d }\times \text{ V}}$$

where d is the dilution factor, and V is volume of inoculum (mL).

#### Composition of Stuart’s Broth (SB)

To obtain Michaelis–Menten kinetics, the concentration of urea in SB was varied (between 1 mM and 3 M), all other components remained constant as 4.75 g/L dipotassium hydrogen phosphate (Merck, Germany), 4.55 g/L potassium dihydrogen phosphate (Merck, Germany), 0.05 g/L yeast extract (Merck, Germany), and 0.005 g/L phenol red (Merck, Germany).

### Enzyme assays

#### Preparation of compounds

Compounds were dissolved in milliQ water (500 mL) and sterilized via syringe filtration (Millipore, 0.22 μm pore size).

#### Colorimetric urease quantification

To access the activity of urease, a colorimetric assay developed by Onal Okyay and Frigi Rodrigues, was used^66^. In the presence of urea, SB undergoes a color change from yellow (Abs_max_ 430 nm) to pink (Abs_max_ 560 nm) occurs. Three biological repeats were completed using a microplate reader (BMG Labtech) with an inbuilt injector system to analyze initial rate of color change.

#### Michaelis–Menten Plots & Dixon Plot

Kinetic assays were prepared by adding 100 μL SB (minus urea) and 100 μL urease (urease from *C. ensiformis* (Type III powder, 15,000–50,000 units/g (Merck, Germany)) (135 U in PBS, determined above) to a 96-well plate (Corning, New York, USA). To the injector system of the plate reader, SB (plus urea (Merck, Germany)) was added (in excess). Each well was read at 560 nm to achieve cycle time of 6 s, each well was read for a total of ten cycles. The injection system then was replaced with SB containing a different (increasing) concentrations of urea and the plate reader method was run again. To measure parameters in the presence of compound, 100 μL of 2-MA (10 mM, dissolved in SB) was added in place of the original 100 μL of SB.

The Dixon plot was performed with three different concentrations of urea (16.6, 83.3, and 166.5 mM) and with five different concentrations of 2-MA (11.0, 27.4, 54.9, 82.3, and 109.7 mM). The reciprocal of initial rate of reaction was plotted against varying inhibitor concentrations to allow determination of K_i_ and the mechanism of inhibition.

Curve fitting and statistical analysis was performed using GraphPad Prism (version 7, GraphPad Software Inc., San Diego, CA). Kinetic models (Michaelis–Menten) were fitted using non-linear regression and SEM, 95% confidence intervals and R^2^ values were assessed.

#### Measuring IC_50_

To determine IC_50_ the Burt, Galetin and Houston protocol was followed^67^. Urea (20 mg/mL in standard SB) and urease (135 U in PBS) were remained constant. Compounds (2-MA and AHA (Merck, Germany)) were varied from (0–900 mM in PBS) and the degree of color change from yellow to pink, was accessed over a 10 min incubation. Compound solution (100 μL) and urease solution (100 μL) were added to 96-well plate (Corning, New York, USA). SB broth (100 μL) was added to the 96-well plate using the plate reader injection system. Inhibition was monitored at 560 nm for 1 h (60 s cycle time for ten cycles). The enzyme response in absence of compound was defined as 0% inhibition (Max) and SB with no enzyme was maximum inhibition (Min). % Inhibition at each inhibition concentration (x) was calculated according to the equation: $\text{\% Inhibition }= 100 \times \frac{1 - (\text{x }-\text{ Min})}{\text{Max }-\text{ Min}}$. The data was fitted with sigmoidal dose–response curves and IC_50_ determined by non-linear regression using GraphPad Prism Version 7.

### In silico analysis of 2-MA binding

Docking experiments using *S. pasteurii* urease. The crystal structure of *S. pasteurii* urease, 4UBP^34^, was used for docking analysis. The protein was prepared using Flare (Flare 3.0.0, Revision 38710; Cresset, Litlington, Cambridgeshire, UK). A docking grid was determined by a 10 Å radius around the Ni (1) in the active site. Docking was carried out by Flare, the ligands were docked using the ‘accurate but slow’ setting. Docking score was determined by Flare, using the LF Rank Score.

QM modelling of *S. pasteurii* urease active site. The system of *S. pasteurii* urease was prepared based on the crystal structures of AHA inhibited and wild type *S. pasteurii* urease with PDB IDs 4UBP^34^ at 1.55 Å resolution and 2UBP^68^ at 2.00 Å resolution, respectively. Hydrogen atoms were added using the fDynamo computational library of Field et al.^69^. Residues were selected according to Nordlander et al.^41^. Systems were subjected to optimization to minima using the Gaussian16^70^ software using the PM6^71^ semi-empirical Hamiltonian with cut-off atoms replaced by frozen, dummy atoms. Single-point density functional calculations were carried out using the B3LYP functional^47–50^ and SDD basis set^51^ for nickel and 6-31G(d) basis set^52^ for all other atoms. Supplementary Table 2 shows individual calculations. Figures were generated using ChemCraft^43^.

### Testing 2-MA on *P. mirabilis* urease

#### Antimicrobial growth assay

In vitro susceptibility of compounds, 2-MA and AHA, were determined using microbroth dilution method. Compounds stocks were prepared at 200 mM. Overnight cultures were subcultured (1:1000 into LB). Into a 96-well plate, 100 µL of LB was added to every column except inhibitor alone in column A. Serial dilutions of compounds were prepared (10–100 mM). Additional controls were bacteria minus compound and compound minus bacteria. The plate was incubated with shaking (200 rpm) before measurements overnight (37 °C) and bacterial growth was measured as function of absorbance at 600 nm.

#### Inhibitor transmembrane movement assay

To access whether compounds could cross the bacterial membrane and inhibit urease intracellularly, the macroscopic changes in pH were measured. To check that the compounds did not alter the viability of the cells, viability cell counts were used to monitor bacteria growth. *P. mirabilis* and *E. coli* overnight cultures were prepared. Media pH and CFU/mL were measured at the start (0 min) and the end (90 min) of bacteria growth.

#### In vitro bladder models and analysis

Bladder models and artificial urine were prepared according to Nzakizwanayo et al.^56^ (Tables 2 and 3).

**Table 2:** Components 5 × artificial urine, dissolved in 1 L of milliQ water and autoclaved.

Components	Mass (g)
Anhydrous sodium sulphate	11.50
Magnesium chloride hexahydrate	3.25
Sodium chloride	23.00
Tri-sodium citrate	3.25
Sodium oxalate	0.10
Potassium di-hydrogen orthophosphate	14.00
Potassium chloride	8.00
Ammonium chloride	5.00
Gelatine	25.00
Tryptone soya broth	5.00

The pH was adjusted to pH = 5.7 by the addition of sodium hydroxide (all components were purchased from Merck, Germany).

**Table 3:** Components 5 × artificial urine, dissolved in 400 mL of milliQ water.

Components	Mass (g)
Urea	125.00
Calcium chloride	2.45

Urea is added to water which has been gently warmed with stirring to allow it to fully dissolve. The 400 mL of solution is filtered through 0.45 µm syringe filter (Millipore, UK). The two solutions were combined with 3.6 L of sterile milliQ water. The pH of the artificial urine was adjusted to 6.1 before use. Compounds were dissolved in urine reservoirs, "kidney" (2 × 5 L, 10 mM per kidney). Models were used to stimulate a late-stage infection, hence they were inoculated with 10^8^ CFU/mL of *P. mirabilis*. Artificial urine containing the compounds were pumped into the bladders at a constant rate of 0.75 mL/min. The number of viable bacteria and pH were measured at periodic intervals throughout. Bladders were treated with 2-MA, AHA, and no treatment.

#### Crystal violet staining for biofilm analysis

To test whether 2-MA and AHA inhibited biofilm activity, biofilms were prepared as follows. *P. mirabilis* was subcultured (1:1000 into LB, total volume of 1 mL), each subculture was added to a 12-well micro-plate (Corning, New York, USA) thus the starting inoculum was 10^3^ CFU/mL. Microplates were incubated statically with varying concentrations of compound (1, 2.5, 5, 7.5, 10 and 15 mM) for 24 h at 37 °C. To quantify the biofilms crystal violet staining was used, whereby biofilms are washed 3 × with milliQ water removing planktonic cells. The adhered cells were stained with crystal violet solution (0.1% (w/v), 500 μL) and incubated statically for 15 min at RT. The stain was removed with 3 × exhaustive washes with milliQ water and dried at RT for 30 min. Quantification of adhered cells occurred by adding decolorizing solution (33% (v/v) acetic acid, 500 μL) to each well and the absorbance measured at 595 nm. Positive control: absence of compound, negative control: absence of bacteria.

### Cytotoxicity testing

#### Testing cell viability on Mammalian cells

To prepare the mammalian cells, spontaneously transformed aneuploid immortal keratinocytes (HaCaTs) (HaCaTs originated from DKFZ, Heidelberg) were resuscitated from frozen stocks (liquid N_2_) in Dulbecco’s Eagle Medium (DMEM, (Merck, Germany)) supplemented with fetal calf serum (10% (v/v), (Merck, Germany)) and cultured in controlled atmosphere (5% CO_2_, 37 °C). Adherent cells were routinely passaged in T75 culture asks every 2–3 days or when cells had reached 70–80% confluency (low passage number cell lines were used for experimental analysis). HaCaT cells were detached by pre-washing in PBS with EDTA (1 mM, 10 mL, incubated for 12 min in order to chelate divalent cations, (Merck, Germany)), followed by trypsinization (0.25%, 3–4 min). Cells were resuspended in fresh medium (DHEM), counted using a hemocytometer, and the cell solution diluted appropriately with DMEM to achieve 5 × 10^3^ cells per well. Cells were seeded in triplicate into 96-well plate (200 μL) and incubated overnight to form sub-confluent monolayers. XTT assay: after 24 h incubation, half the incubated media (100 μL) was treated with compounds (sterile filtered 0.22 μm pore size (Millipore) in PBS) at 80, 40, 20, 10, 5, 2.5, 1.25, and 0.625 mM to assess concentration-dependent cytotoxicity. A negative control of PBS was prepared for untreated cells. After overnight incubation with the drug, cells were washed (3 × PBS, 200 μL) to remove residual compound and cell proliferation was measured using a standard 2,3-bis[2-methoxy-4-nitro-5-sulphophenyl]-2H-tetrazolium-5-carboxanilide inner salt (XTT) assay kit (CyQUANT, (Fisher Scientific, UK)). The assay was prepared according to manufacturer’s instructions^72^. In brief, XTT-labelling reagent and electron coupling reagent were mixed (ratio 50:1) and added to 96-well plate (50 μL) with fresh DMEM media (100 μL). Plates were incubated for 4 h (37 °C, 5% CO_2_ in a humidified atmosphere). Quantification of cell cytotoxicity was measured as a function of absorbance at 450 nm in a plate reader (BMJ Labtech). The cytotoxic effect of treatment was expressed as % viability compared to untreated cells: $\text{\% cell viability }= \frac{\text{corrected absorbance of sample cells}}{\text{corrected absorbance of untreated cells}}\text{ x }100$. The background correct was blank (media only). Cells treated with Triton X-100 (20% (v/v) (Merck, Germany)) were used as positive control for cytotoxic effect (treated for the same time as the compounds). Each experiment had three biological repeats.

#### Ex vivo hemolytic assay

This project has been approved by the University of Bath, Research Ethics Approval Committee for Health (REACH) [reference: EP 18/19 108]. Informal consent was gained from the donor prior to research taking place, and research was carried out following the relevant guidelines defined by REACH. Whole blood (25 mL) was obtained from an anonymous human donor, drawn directly into lithium heparin-coated Vacutainer tubes to prevent coagulation. Blood was centrifuged (797*g*, 10 min, 5810 R Eppendorf, Stevenage, UK), levels of hematocrit and plasma were marked on the tube. Plasma layer was removed, discarded, and hematocrit tube was filled to the volume of plasma with saline solution (150 mM NaCl (Merck, Germany)). Blood cells were washed three times (centrifugation, aspiration of supernatant, and replacement with fresh saline). Pellet containing erythrocytes were resuspended in PBS and divided into 500 μL aliquots. Aliquots were centrifuged (345 g, 10 min, Eppendorf 5418 R, Stevenage, UK), and the supernatant was replaced with 500 μL of varying concentrations (40, 20, 10, 5, 2.5, 1.25, 0.625 mM) of syringe filtered (0.22 μm pore size, Millipore) compounds. Cells were incubated with compounds for 30 min (37 °C, with agitation) before being centrifuged (as before), the supernatant was removed to measure the absorbance of liberated hemoglobin at 415 nm, as described by Tramer et al*.*^73^. Total lysis of erythrocytes was obtained by treatment with Triton X-100 (20% (v/v)). Degree of hemolysis was expressed as % hemolysis, relative to spontaneous lysis controls. $\text{\% }haemolysis = \frac{\text{corrected absorbance of sample cells}}{\text{corrected absorbance of lysed cells}} \times 100$. Values were background correct to blank (PBS only). Each experiment had three biological replicates.

## Supplementary Information

Supplementary Information.
