# Supplementary material for: A small-molecular inhibitor against Proteus mirabilis urease to treat catheter-associated urinary tract infections
Source: Sci Rep. 2021 Feb 12;11:3726. doi: 10.1038/s41598-021-83257-2 (PMC7881204; doi:10.1038/s41598-021-83257-2)
Supplement: Supplementary file 1 — Supplementary Information. [file 41598_2021_83257_MOESM1_ESM.docx]

**A Small-Molecular Inhibitor against *Proteus mirabilis* Urease to treat Catheter-Associated Urinary Tract Infections**

**Scarlet Milo,^1+^ Rachel A. Heylen,^1+^ John Glancy,^1^ George T. Williams,^2^ Bethany L. Patenall^1^, Hollie J. Hathaway,^3^ Naing T. Thet,^1^ Sarah L. Allinson,^4^ Maisem Laabei,^5^ and A. Toby A. Jenkins^1^***

^1^ Department of Chemistry, University of Bath, Bath, BA2 7AY, UK.

^2^ School of Physical sciences, University of Kent, Canterbury, CT2 7NH, UK.

^3^ Department of Chemistry, Lancaster University, Bailrigg, Lancaster, LA1 4YB, UK.

^4^ Biomedical and Life Sciences Division, Lancaster University, Bailrigg, Lancaster, LA1 4YB, UK.

^5^ Department of Biology and Biochemistry, University of Bath, Bath, BA2 7AY, UK.

^+^ these authors contributed equally to this work

*email: [A.T.A.Jenkins@bath.ac.uk](mailto:A.T.A.Jenkins@bath.ac.uk)


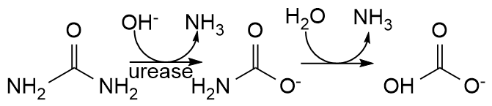
**Supplementary Information:**

**Supplementary Figure 1.** Urease catalyzed hydrolysis of urea. Accumulation of ammonia within urine of catheterized patients leads to rapid and significant urine alkalization

**
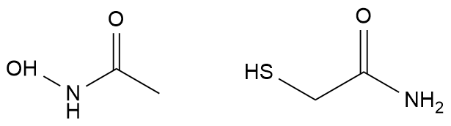
**

**Supplementary Figure 2.** Molecular structure of acetohydroxamic acid, licensed urease inhibitor.

**Supplementary Table 1:** Michaelis-Menten parameters for *C. ensiformis* urease, in the presence and absence of 2-MA.

|  | K_M_ (M) ± SEM (M) | V_max_ (M) ± SEM (M) |
| --- | --- | --- |
| - 2-MA | 0.2 ± 0.0299 | 1.58 x 10^-2^ ± 7.70 x10^-4^ |
| +2-MA | 8.27 ± 6.07 | 9.56 x 10^-3^ ± 6.03 x 10^-3^ |

**
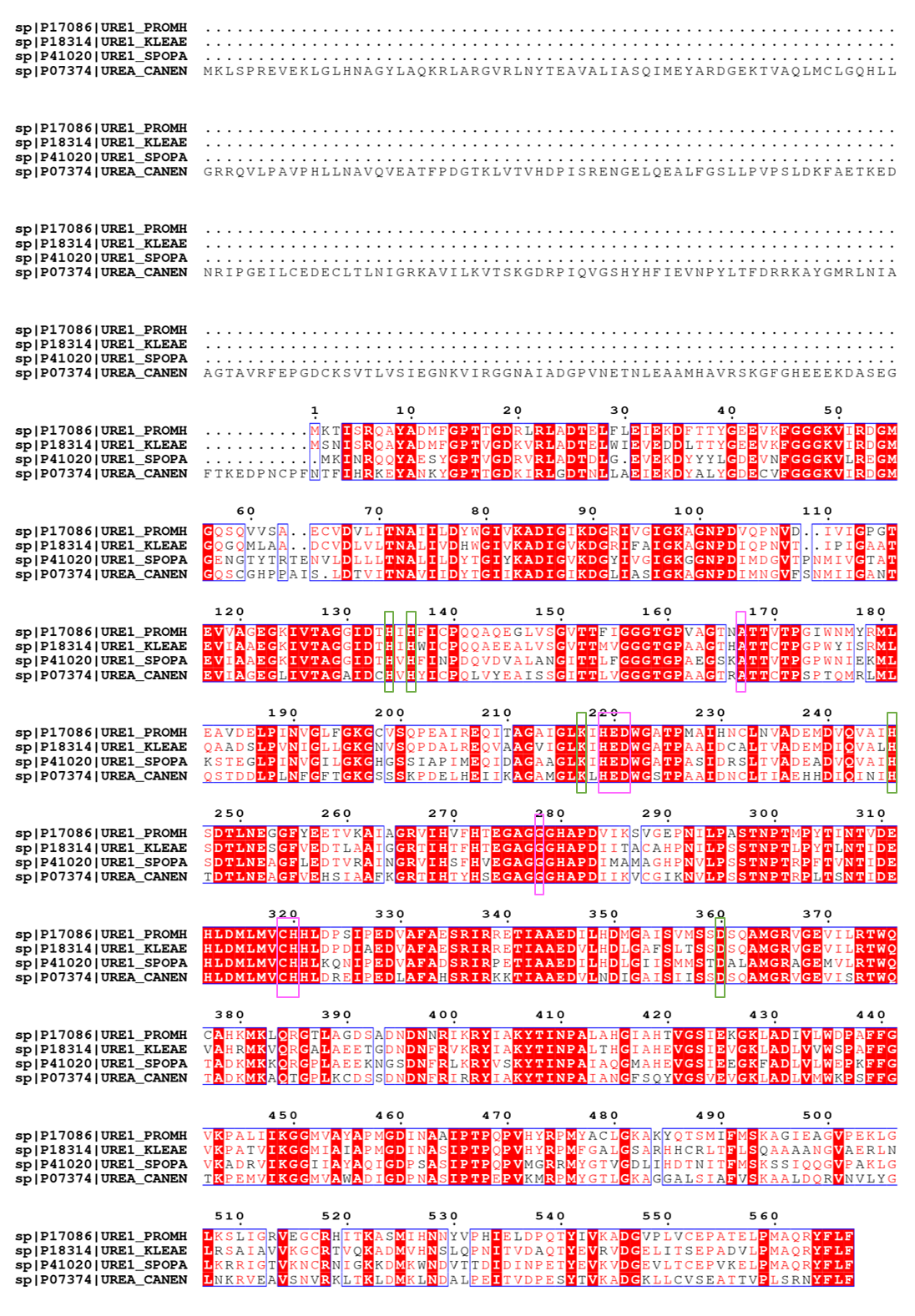
**

**Supplementary Figure 3.** An alignment of the protein sequences for alpha subunit of urease. P17086 is the sequence from *Proteus mirabilis* (strain HI4320); P18314 is from *Klebsiella aerogenes*; P41020 is from *Sporosarcina pasteurii* (formly known as *Bacillus pasterii*); and P07374 is from *Canavalia ensiformis*. Sequences were obtained from the Uniprot database^1^. The green boxes identify amino acids important in coordinating the Ni atoms in the active site. Those amino acids identified by pink boxes are also involved in the catalytic mechanism ^2^. Alignment was carried out using Multalin^3^ and presented using ENDscript^4^.

**Supplementary Table 2.** Calculated energies for species involved in ligand binding to *S. pasteurii* urease active site. Wild-type (WT) active site structure involves the common architecture of the structures in Fig. 3A-F, one bridging hydroxide, one water molecule coordinated to Ni(1) and one water molecule coordinated to Ni(2). Binding energies are calculated by subtraction of WT and relevant neutral ligand’s potential energy from sum of the active site structure and liberated water molecules’ potential energy.

|  | Potential energy [E_h_] | | | Binding energy PM6 | | Binding energy B3LYP/6-31G(d)/SDD | |
| --- | --- | --- | --- | --- | --- | --- | --- |
|  | PM6 | RB3LYP | UB3LYP | [E_h_] | [kJ mol^–1^] | [E_h_] | [kJ mol^–1^] |
| Small molecules optimised in PCM solvent = water | | | | | | | |
| H_2_O | -0.0929664 | -76.41591904 | - | - | - | - | - |
| AHA neutral | -0.1097123 | -284.3704716 | -284.3704716 | - | - | - | - |
| 2-MA neutral | -0.0936801 | -607.4000371 | -607.4000371 | - | - | - | - |
| Urea neutral | -0.0934248 | -225.2709437 | - | - | - | - | - |
| Active site structures optimised *in vacuo* | | | | | | | |
| WT | -0.2029055 | - | -3217.385535 | - | - | - | - |
| 7A | -0.2040011 | - | -3366.270167 | -0.0006372 | -1.6729686 | -0.029607095 | -77.73342713 |
| 7B | -0.1895452 | - | -3748.394245 | 0.014074 | 36.951287 | -0.024591756 | -64.56565459 |
| 7C | -0.1530688 | - | -3289.816086 | -0.0426713 | -112.0334982 | 0.008554831 | 22.46070774 |
| 7D | -0.1497457 | - | -3671.955826 | -0.0390929 | -102.638409 | -0.00209174 | -5.491864421 |
| 7E | -0.0630197 | - | -3272.511365 | -0.0293011 | -76.93003805 | -0.00311596 | -8.180953242 |
| 7F | -0.0463804 | - | -3595.479511 | -0.028694 | -75.336097 | 0.058304185 | 153.0776375 |

**Supplementary Figure 4.** Determining effect of 2-MA **(a)** and AHA **(b)** on the growth of *P. mirabilis* strain B4. 10 mM was determined to be the maximum tolerable concentration of both 2-MA and AHA. Results are from three biological replicates, error bars calculated using SEM. Data fitted using GraphPad Prism Version 7.

**a**

**b**


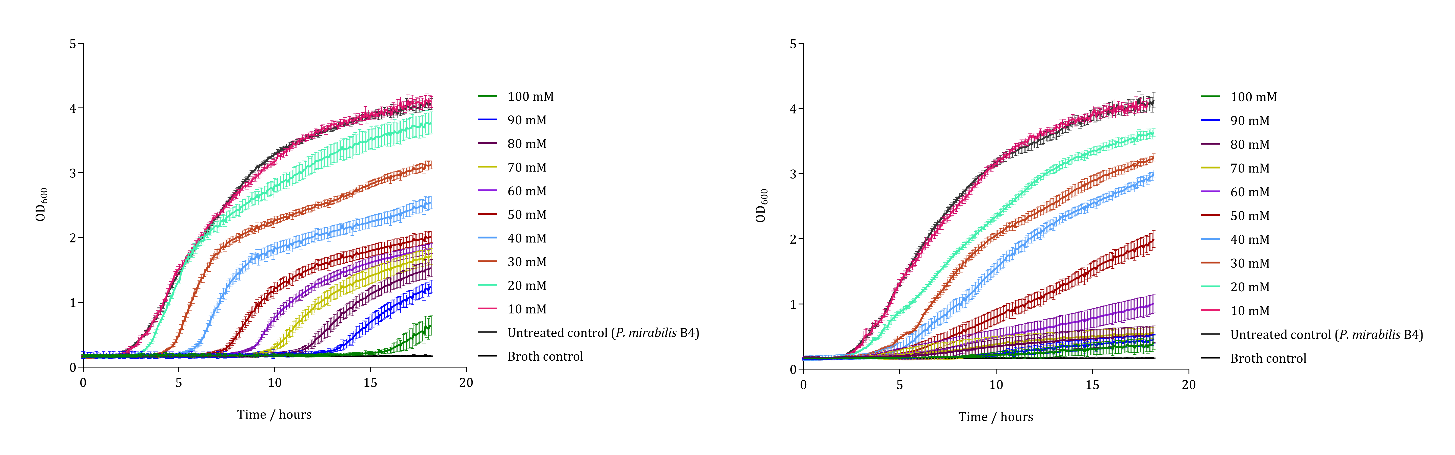


**
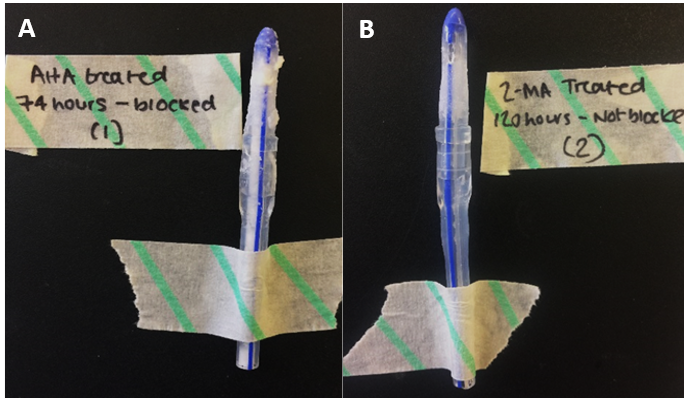
Supplementary Figure 5.** Images of the catheter tips post in vitro bladder model experiment. **(a)** Catheter taken from the AHA treated model, shows extensive encrustation of the catheter tip at the time of blockage (74 hours). Thus, the crystal deposits had blocked the catheter eyelet and lumen. **(b)** Catheter taken from 2-MA treated model (120 hours). This catheter did not block throughout the duration of the experiment.

**
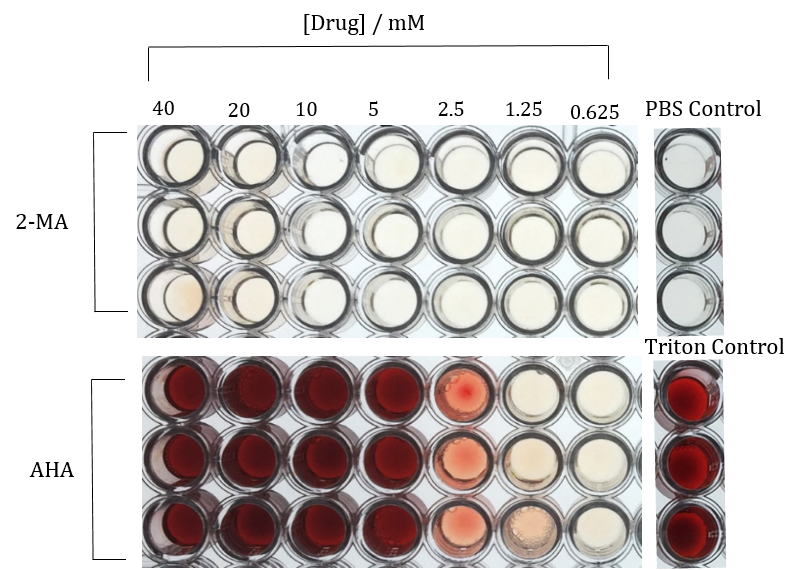
Supplementary Figure 6.** Image of the haemolysis analysis comparing AHA with 2-MA. Measurement at 410 nm, followed by correction with negative control and calculation of % haemolysis using Triton control indicated a significant difference, *p* < 0.0001, between 2-MA and AHA at all concentrations (Fig. 7b). Visual analysis of the haemolysis of plate also indicates the difference in AHA and 2-MA to cause haemolysis

.


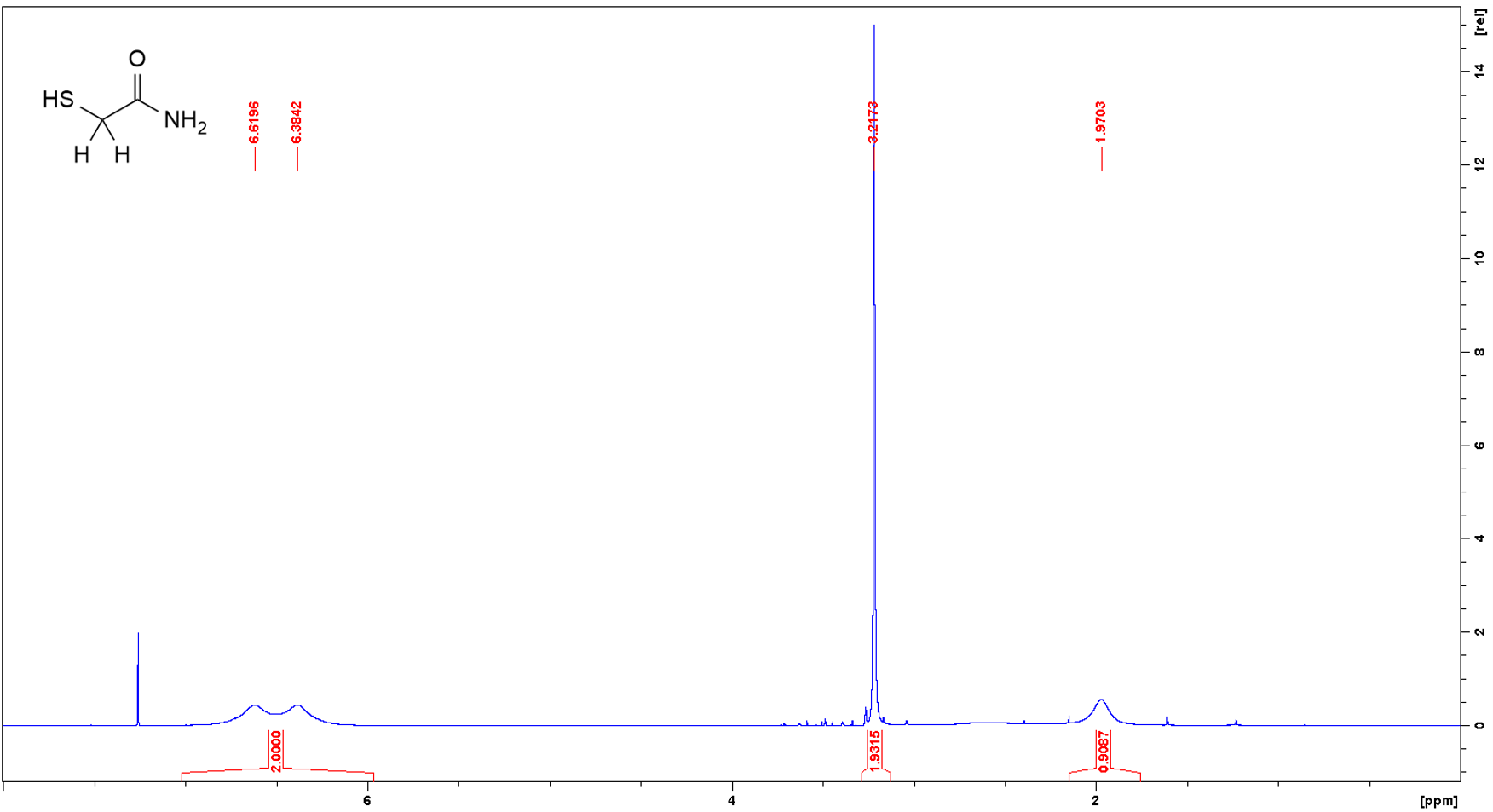


**Supplementary Figure 7.** ^1^H NMR of 2-mercaptoacetamide. ^1^H NMR (Bruker 400 MHz, CDCl_3_) δ: 1.97 (s, 1H, SH), 3.22 (s, 2H, CH_2_), 6.38 (br, 1H, NH_2_), 6.62 (br, 1H, NH_2_). This matches 1H NMR described by Bockman *et al.,*^5^.

**References:**

1. Consortium, T. U. UniProt: A worldwide hub of protein knowledge. *Nucleic Acids Res.* **47**, D506–D515 (2019).

2. Benini, S. *et al.* The complex of Bacillus pasteurii urease with acetohydroxamate anion from X-ray data at 1.55 Å resolution. *J. Biol. Inorg. Chem.* **5**, 110–118 (2000).

3. Corpet, F. Multiple sequence alignment with hierarchical clustering. *Nucleic Acids Res.* **16**, 10881–10890 (1988).

4. Robert, X. & Gouet, P. Deciphering key features in protein structures with the new ENDscript server. *Nucleic Acids Res.* **42**, 320–324 (2014).

5. Bockman, M. R. *et al.* Investigation of (S)-(-)-acidomycin: A selective antimycobacterial natural product that inhibits biotin synthase. *ACS Infect. Dis.* **5**, 598–617 (2019).
